# Initial development of Supportive care Assessment, Prioritization and Recommendations for Kids (SPARK), a symptom screening and management application

**DOI:** 10.1186/s12911-018-0715-6

**Published:** 2019-01-10

**Authors:** Sadie Cook, Emily Vettese, Dilip Soman, Shannon Hyslop, Susan Kuczynski, Brenda Spiegler, Hailey Davis, Nathan Duong, Stacee Ou Wai, Robert Golabek, Patryk Golabek, Adam Antoszek-Rallo, Tal Schechter, L. Lee Dupuis, Lillian Sung

**Affiliations:** 1 Program in Child Health Evaluative Sciences, The Hospital for Sick Children, Peter Gilgan Centre for Research and Learning, 686 Bay Street, Toronto, Ontario M5G 0A4 Canada; 2 Rotman School of Management, University of Toronto, 105 St. George Street, Toronto, Ontario M5S 3E6 Canada; 3 Ontario Parents Advocating for Children with Cancer (OPACC), 99 Citation Drive, Toronto, Ontario M2K 1S9 Canada; 4 Department of Psychology, The Hospital for Sick Children, 555 University Avenue, Toronto, Ontario M5G 1X8 Canada; 5 Translucent Computing, 1 Yonge Street, Toronto, Ontario M5E 1E5 Canada; 6 Catalyst Workshop Inc, 192 Spadina Avenue, Suite 108, Toronto, Ontario M5T 2C2 Canada; 7 Division of Haematology/Oncology, The Hospital for Sick Children, 555 University Avenue, Toronto, Ontario M5G 1X8 Canada; 8 Department of Pharmacy, The Hospital for Sick Children, 555 University Avenue, Toronto, Ontario M5G 1X8 Canada

**Keywords:** Pediatric cancer, Symptom screening, Supportive care, Self-report, Oncology, Website development

## Abstract

**Background:**

We developed Supportive care Prioritization, Assessment and Recommendations for Kids (SPARK), a web-based application designed to facilitate symptom screening by children receiving cancer treatments and access to supportive care clinical practice guidelines primarily by healthcare providers. The objective was to describe the initial development and evaluation of SPARK from the perspective of children.

**Implementation:**

Development and evaluation occurred in three phases: (1) low fidelity focused on functionality, (2) design focused on “look and feel” and (3) high fidelity confirmed functionality and design. Cognitive interviews were conducted with children receiving cancer treatments 8–18 years of age. Evaluation occurred after every five interviews and changes were guided by a Review Panel. Quantitative evaluation included SPARK ease of use and understandability of SPARK reports.

**Results:**

The number of children included by phase were: low fidelity (*n* = 30), design (n = 30) and high fidelity (n = 30). Across phases, the median age was 13.2 (range 8.5 to 18.4) years. During low-fidelity and design phases, iterative refinements to SPARK improved website navigation, usability and likability from the perspective of children and established symptom report design. Among the last 10 children enrolled to high-fidelity testing, all (100%) understood how to complete symptom screening, access reports and interpret reports. Among these 10 respondents, all (100%) found SPARK easy to use and 9 (90%) found SPARK reports were easy to understand.

**Conclusions:**

SPARK is a web-based application which is usable and understandable, and it is now appropriate to use for research. Future efforts will focus on clinical implementation of SPARK.

## Background

Children receiving cancer treatments have excellent survival outcomes, in part, related to the provision of intensive therapies. Unfortunately, most children suffer and experience severe and bothersome treatment-related symptoms [1]. Common symptoms include pain, mouth sores, nausea, fatigue, sadness and worry [2]. Symptoms remain unaddressed even during healthcare encounters because children do not complain and clinicians fail to ask about them. Systematic screening of symptoms and easy access to evidence-based interventions to address bothersome symptoms are key to the delivery of optimal supportive care in pediatric oncology [3].

**Fig. 1:**
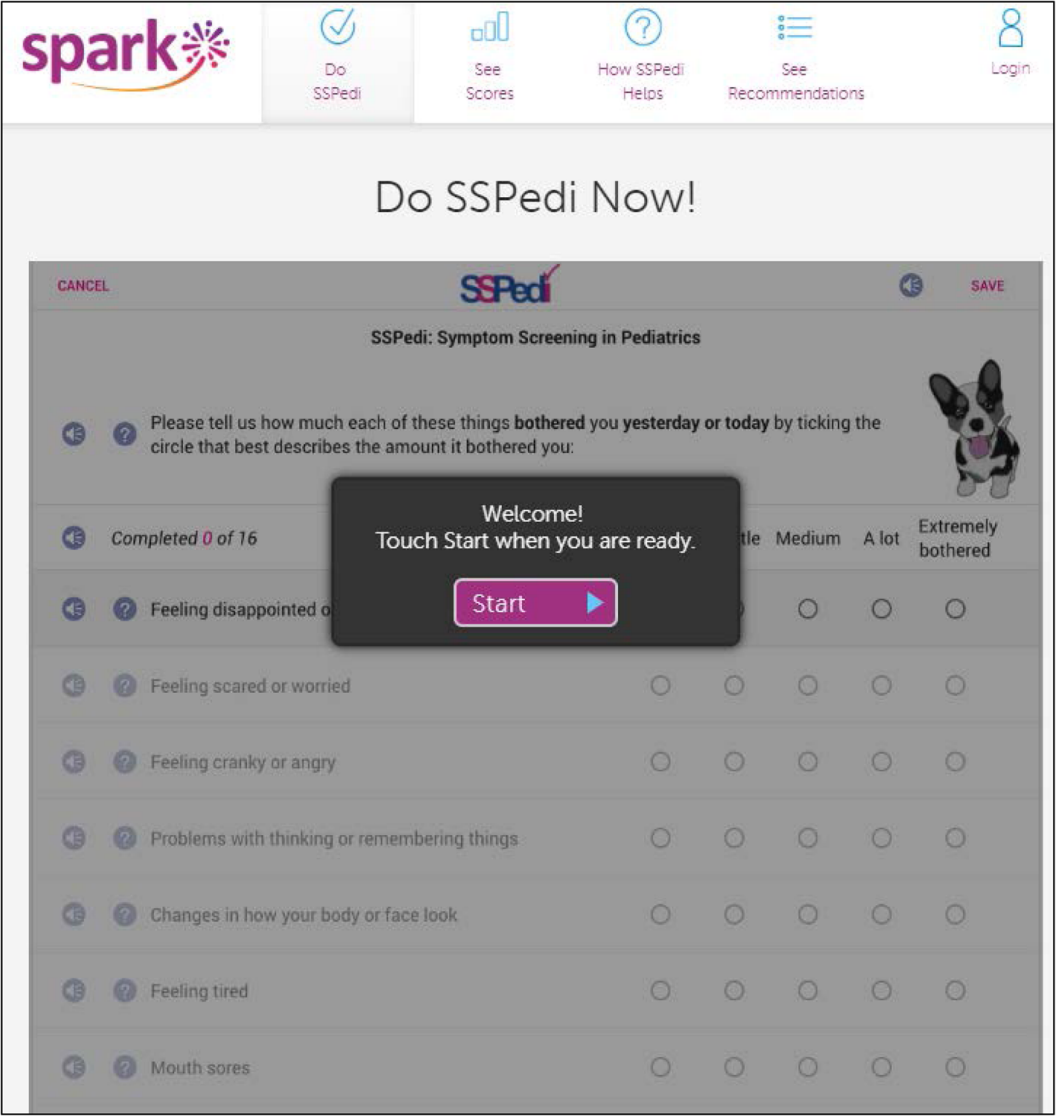
Symptom Screening in Pediatrics Tool (SSPedi)

In order to facilitate systematic symptom screening, a tool appropriate for children with cancer is required [4]. We thus developed the Symptom Screening in Pediatrics Tool (SSPedi), a 15-item paper or electronic tool that asks children receiving cancer treatments how much each symptom bothered them yesterday or today [5–8]. The electronic version of SSPedi has features specifically designed to facilitate child self-report and children find it easy to use (Fig. 1) [7]. The tool uses a 5-point Likert scale ranging from “not at all bothered” to “extremely bothered”. SSPedi has excellent psychometric properties for child self-report and guardian proxy-report administration [8, 9]

.

In addition to the development and evaluation of SSPedi, we also appreciated the need to facilitate symptom management through improving access to clinical practice guidelines for supportive care needs. Clinical practice guidelines are recommendations based on systematic reviews of the evidence and an assessment of the benefits and harms of different strategies that aim to improve patient care [10]. Guideline-consistent care has been shown to improve patient outcomes in several areas including oncology [11–14].

In order to bring symptom screening and access to clinical practice guidelines together toward improving the supportive care of children with cancer and pediatric hematopoietic stem cell transplantation recipients, we developed Supportive care Prioritization, Assessment and Recommendations for Kids (SPARK). SPARK is a web-based application that consists of two components: (1) a symptom screening component centered on SSPedi, and (2) a supportive care clinical practice guideline component. SPARK tracks symptoms over time, allows patients, family members and health care professionals to view symptom reports, and facilitates access to supportive care clinical practice guidelines. For SPARK to have clinical utility, it has to be useable and liked from the perspective of the intended users. The objective was to describe the initial development and evaluation of the symptom screening aspect of SPARK from the perspective of children receiving cancer treatments.

## Implementation

### Overview

**Fig. 2:**
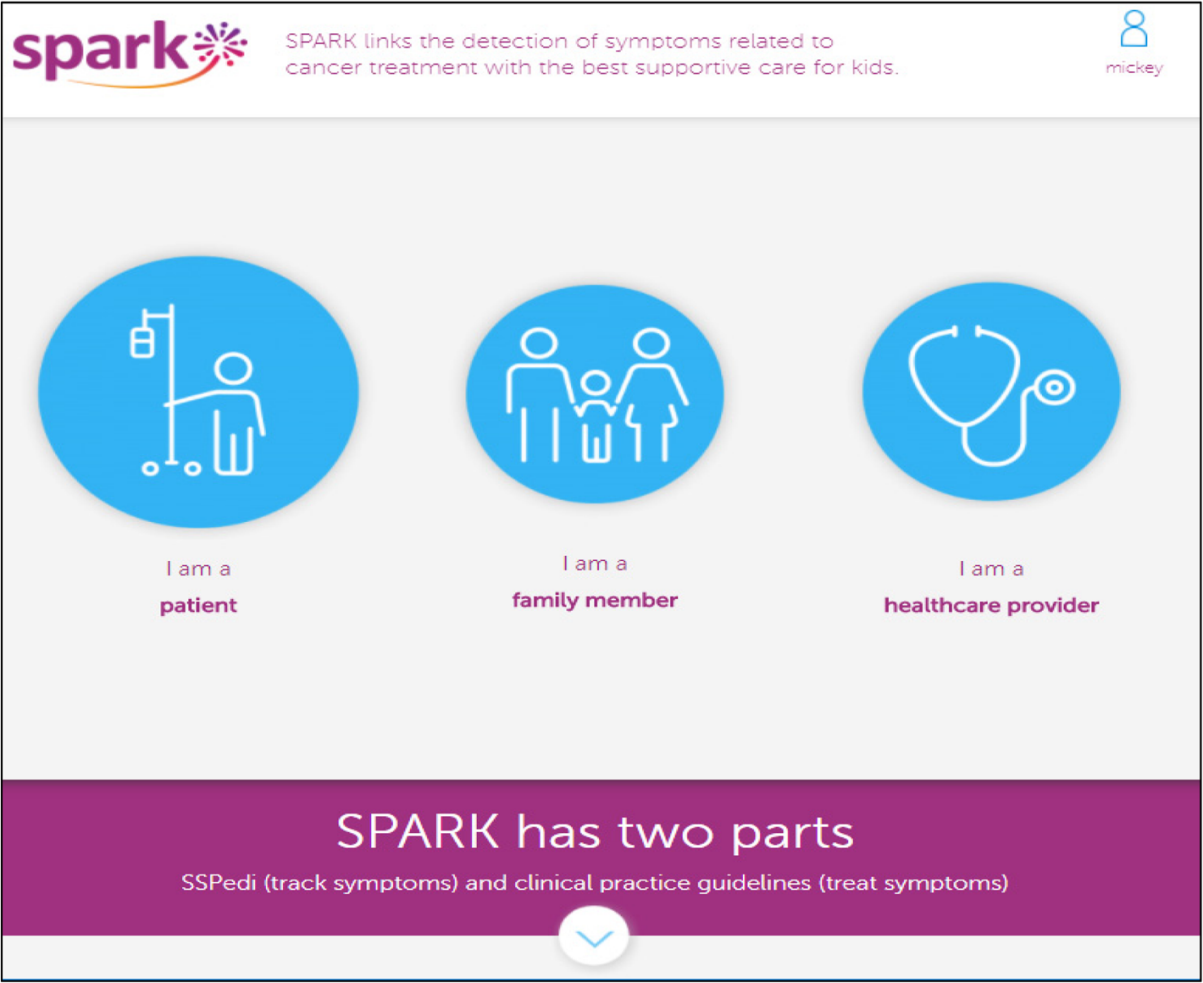
SPARK Landing Page

**Fig. 3:**
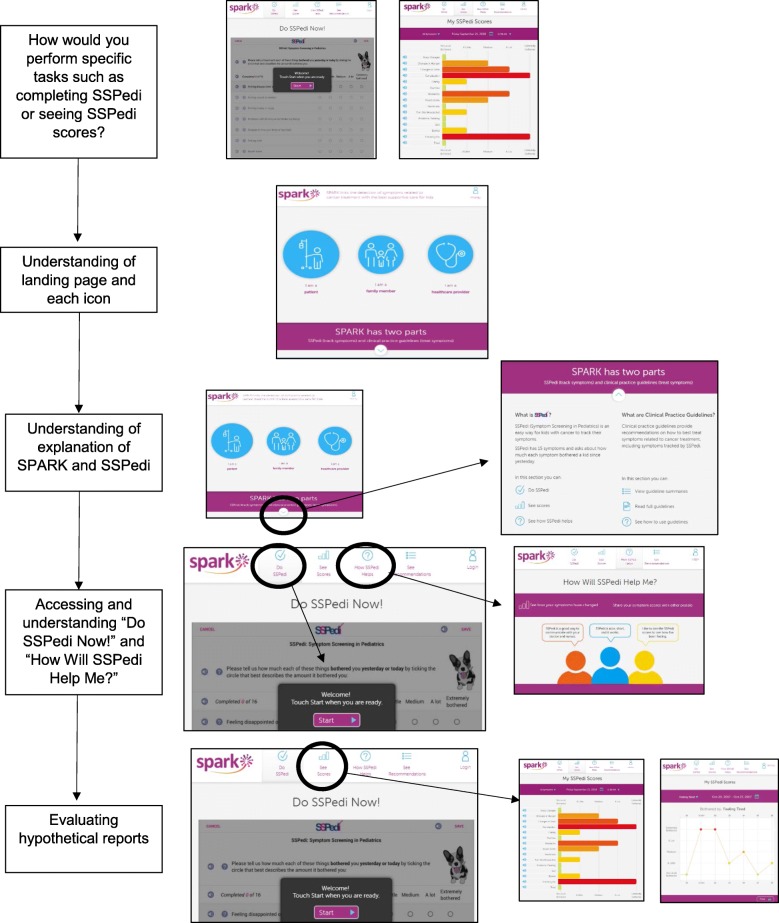
General Flow of the Participant Interview

SPARK pages were initially developed by the research team and a web application development company, Translucent Computing Inc. While SPARK is designed for use by pediatric patients, family members and healthcare providers, this study focused on pages aimed at pediatric patients (or the patient-facing SPARK portal). These pages were: (1) the landing page (Fig. 2), (2) access to SSPedi (“Do SSPedi Now!”) (Fig. 1), (3) information about how SSPedi helps (“How Will SSPedi Help Me?”), and (4) reports (“My SSPedi Scores”). SPARK also facilitates access to supportive care clinical practice guidelines. These guidelines are currently directed at healthcare providers, with development of recommendations for family members and pediatric patients as a future goal. The clinical practice guideline aspect of SPARK will not be described further in this manuscript.

The landing page (Fig. 2) includes three icons: one for patients, one for family members and one for healthcare providers. Only the pages accessed through the patient icon on the landing page were evaluated in this study. When the patient icon is chosen, the “Do SSPedi Now!” page appears (Fig. 1). When pediatric patients reach this page, they are encouraged to complete SSPedi at that moment. From the navigation bar at the top, children can access “How will SSPedi Help Me?” and “My SSPedi Scores”. “How Will SSPedi Help Me?” contains testimonials from children interviewed during SSPedi development who spoke about the benefits of completing SSPedi. “My SSPedi Scores” allows users to see their previous SSPedi scores and trends over time for a specific symptom or for the total SSPedi score. The total SSPedi score ranges from 0 to 60 with higher numbers reflecting more symptom bother.

Development and evaluation of SPARK occurred in 3 phases: (1) low fidelity, (2) design and (3) high fidelity. The main purpose of the low-fidelity phase was to evaluate and enhance the functionality of SPARK web pages, concentrating on how to access and complete SSPedi, navigate between different pages and view reports. All pages were in black and white (grey scale). The main purpose of the design phase was to attain the desired “look and feel” of SPARK. All pages were in color. The design phase was divided into two sub-phases, namely SSPedi report design and overall design. The main purpose of the high-fidelity phase was to evaluate and confirm both functionality and design. This phase was divided into two sub-phases, namely coding sprints and finalization. The phases and sub-phases are detailed under Procedures below.

### Theoretical background

We used the Technology Acceptance Model as the guiding principle behind this research. This model states that perceived ease of use and perceived usefulness are predictors of behavioral intention and actual use of health technology [15]. Consequently, we focused on evaluating these domains during SPARK development.

### Participants

We included English-speaking children and adolescents with cancer and pediatric hematopoietic stem cell transplant recipients who were 8–18 years of age at the time of enrollment. Exclusion criteria were illness severity, cognitive disability or visual impairment that precluded utilization of SPARK according to the primary healthcare team. Children were purposively sampled to maximize variation by age group, gender and underlying diagnosis. All participants were recruited from The Hospital for Sick Children in Toronto, Canada.

### Procedures

For each phase, both quantitative and qualitative data were collected and used to make decisions about development. Draft versions of SPARK were reviewed and approved by a Review Panel composed of two pediatric oncology survivors (ND and HD), one parent advocate (SK), a pediatric psychologist (BS), a behavioral scientist (DS), a pediatric pharmacist (LD) and a pediatric oncologist (LS). Behavioral science was used as an input into choice architecture, which is the process of designing information and choice context to nudge individuals toward making better decisions [16].

In general, participants were first given the opportunity to explore the website freely and then asked to complete specific tasks. The think aloud method was incorporated within the interviews to evaluate different aspects of SPARK [17]. Participants were asked to verbalize their thought process as they navigated through SPARK and were continually prompted to think aloud. An interview guide was used to ensure consistency in the approach. The interviewers were trained clinical research associates with expertise in qualitative approaches and cognitive interviewing. The interview guide began by asking the participant to explore a specific section of the website and to explain what they were seeing and their reactions during this navigation process. This general approach was then followed by specific questions asking the participant to complete specific tasks or their understanding of specific elements. A second individual observed all interviews so that understandability of each aspect could be rated. The second interviewer also recorded field notes. No videos or audio-recordings were made of interviews. Figure 3 outlines the general flow of the participant interview. Details of the specific phases are as follows.

#### Low-Fidelity phase (cohorts 1–3)

The purpose of this phase was to evaluate and enhance the functionality or usability of SPARK web pages. All pages were in black and white (grey scale) to determine whether participants understood how to use SPARK without distraction by color, since addition of color would introduce preferences for a specific design.

**Fig. 4:**
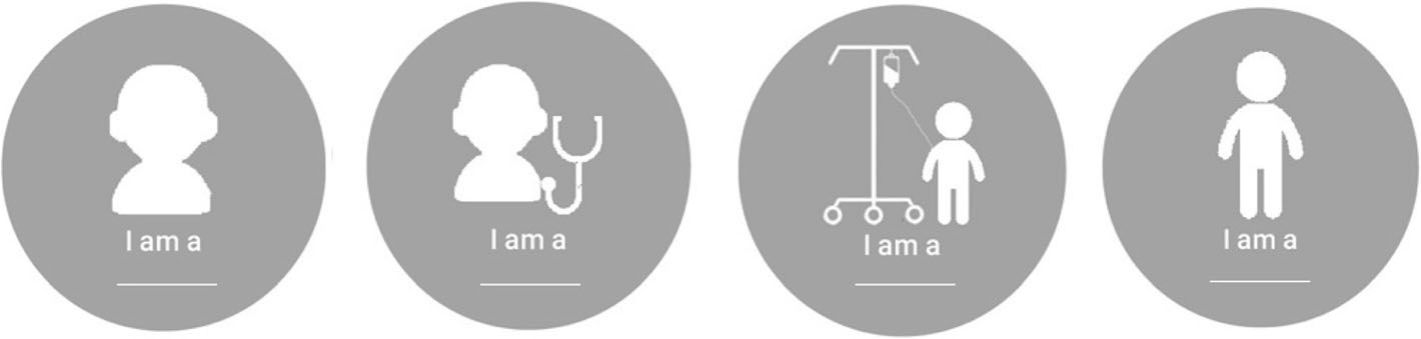
Patient Icons Shown in the Low-Fidelity Phase

First, we had to identify an appropriate patient icon and terminology to refer to patients. Thus, for the first 20 participants, we identified preference for the patient icon among four choices (Fig. 4); we randomized the order in which each icon was shown. Next, we asked participants how they would like to be referred to with the icon they chose. We elicited their preferred term spontaneously and then asked whether any of the following terms were disliked or upsetting: patient, kid, child, young person or teenager.

To make it clear what sections were specifically evaluated for understanding, the “label” used in the Results follows the task description in bold and parenthesis. Low-fidelity evaluation was conducted using a mock version of the website that was created by uploading an image of each page to InVision (https://www.invisionapp.com/). Clickable areas on each image mimicked that of a functional website. We gave participants time to navigate through the different patient-specific web pages of SPARK. After free-exploring, we asked participants how they would perform specific tasks such as completing SSPedi **(Completing SSPedi)** or seeing their current or previous SSPedi scores **(Seeing current or previous SSPedi score)**. Next, we evaluated understanding of each patient-specific page and asked what each page, element and icon meant. From the landing page, we evaluated understanding of the overall page **(Landing Page – Overall)** and each of the child, family member and healthcare provider icons **(Landing Page – Child, Family member and Healthcare provider icons)**. If the user scrolls to the bottom of the landing page, there is an explanation of SPARK and SSPedi; this section was also evaluated **(What are SPARK and SSPedi)**. We then evaluated understanding of the “Do SSPedi Now!” page **(Do SSPedi Now!)** and the “How Will SSPedi Help Me?” page **(How Will SSPedi Help Me?)**.

**Fig. 5:**
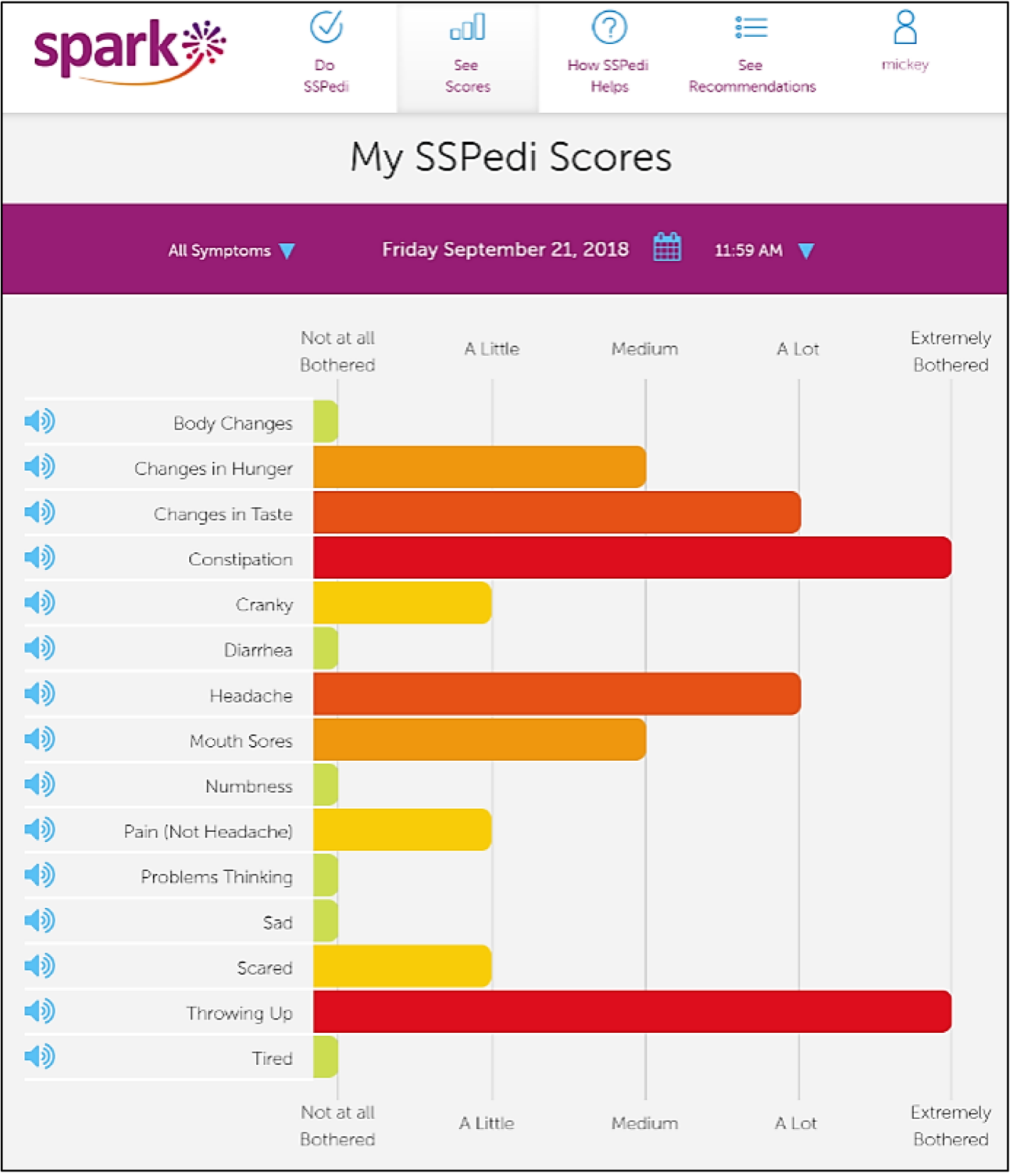
High-Fidelity Phase Version of Single SSPedi Administration Report

From the “My SSPedi Scores” page, we asked participants to evaluate three hypothetical reports: (1) single SSPedi administration showing degree of bother of 15 symptoms (**Single SSPedi administration report**, Fig. 5); (2) specific symptom longitudinally with improvement over time (**Interpreting specific symptom improving over time**); and (3) specific symptom longitudinally with worsening over time (**Interpreting specific symptom worsening over time**). Participants were asked to interpret the vertical and horizontal axes, find specific scores on a specific date (for the longitudinal reports) and to interpret each report.

Finally, we asked questions about the website overall regarding the ease of use of SPARK, ease of understandability of reports and usefulness for future children receiving cancer treatments.

#### Design phase (cohorts 4–7)

During the design phase, pages were modified to attain the desired “look and feel”. The design company Catalyst Workshop Inc. collaborated with Translucent Inc. and the research team to complete this phase. Color was incorporated. The design phase was divided into two sub-phases, namely SSPedi report design and overall design. The SSPedi report design sub-phase (cohorts 4 and 5) focused on how best to display a single SSPedi administration report (Fig. 5), concentrating on bar graph orientation and color scheme. We presented different options concurrently and asked respondents to choose their preferred option. More specifically, we evaluated whether respondents preferred to see symptom scores horizontally or vertically and whether they preferred degree of bother scores to be one color or multi-colored. In addition to asking their preferred option, we also asked which option was easier to understand.

Once the SSPedi report design sub-phase was completed, the “look and feel” of this page was used to draft four additional pages in color (cohorts 6 and 7). The additional pages were the landing page, access to SSPedi, information about how SSPedi helps, and trends over time graphs showing a specific symptom (throwing up). We assessed understanding and preferences of pages and elements such as icons, pictures and colors.

#### High-fidelity phase (cohorts 8–10)

The final phase was high fidelity, in which both functionality and design were evaluated and confirmed. Procedures mirrored those of low fidelity except that the live SPARK website was evaluated instead of the mock InVision version. This phase was divided into two sub-phases, namely coding sprints and finalization. Coding sprints (cohorts 8 and 9) focused on a sub-set of SPARK pages and ensured that respondents could access SSPedi, complete SSPedi, retrieve instructions, cancel out of SSPedi and save SSPedi scores. The last sub-phase was finalization (cohort 10) and it evaluated the entire patient-facing portal of SPARK. As with low-fidelity evaluation, we asked questions about the website overall including ease of use of SPARK, ease of understandability of reports, and usefulness for future children receiving cancer treatments.

### Evaluation

Throughout each phase, we evaluated responses after every five interviews and a smaller research team (clinical research associates and principal investigators) met to decide whether the script or SPARK required minor edits. After every 10 to 20 interviews and when the smaller research team was satisfied that a phase had been completed (low fidelity, design and high fidelity), the Review Panel met and could request further edits (which would lead to additional interviews) or confirm completion of a phase.

Quantitative evaluation in low- and high-fidelity phases included understandability, usability, ease of understanding of SPARK reports and usefulness for future children receiving cancer treatments. Understandability was rated externally by experts (two interviewers) while usability, ease of understanding of SPARK reports and usefulness were rated by the user-patient (children themselves). More specifically, understandability of SPARK elements were evaluated by the two interviewers on a 4-point Likert scale ranging from 1 = “completely incorrect” to 4 = “completely correct”. The two interviewers rated understandability independently and then compared ratings. If they disagreed, they referred to field notes to arrive at consensus. Usability of the SPARK website was evaluated by the participant on a 5-point Likert scale ranging from 1 = “very hard” to 5 = “very easy”. Ease of understanding of SPARK reports was similarly rated. Usefulness for future children receiving cancer treatments was evaluated by the participant on a 5-point Likert scale ranging from 1 = “not useful at all” to 5 = “very useful”. We reported the proportion of participants who were correct or completely correct, found SPARK/SPARK reports easy or very easy to use or understand and thought SPARK would be useful or very useful for future children receiving cancer treatments.

Criteria for completion of low-fidelity and high-fidelity (finalization) phases were based upon quantitative and qualitative responses as follows: (1) saturation (absence of new themes); (2) at least 9 of the last 10 participants stated the website was easy or very easy to use; and (3) qualitative comments did not suggest that further modifications were required. Based upon previous experience, we anticipated conducting between 6 and 12 iterations with 5 participants per iteration for a total of 30 to 60 participants for each phase, or 90 to 180 total interviews.

## Results

### Patient participation by phase and icon/label preference

**Fig. 6:**
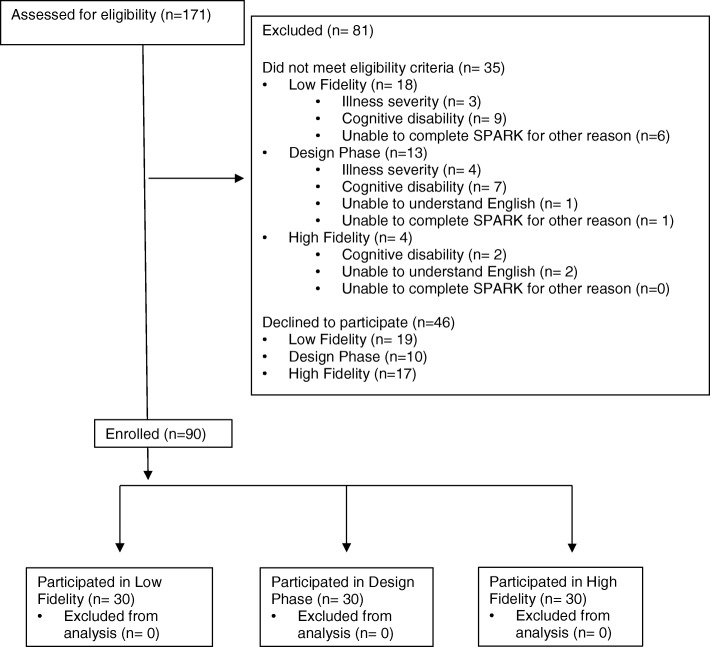
Flow Diagram of Patient Identification and Participation by Phase

Figure 6 shows the flow diagram of patient identification and participation by phase. Overall, 90 children participated with a median age of 13.2 (range 8.5 to 18.4) years; demographic information is shown in Table 1. During low-fidelity development, when we asked the first 20 patients for their preference between the four patient icons (Fig. 4), 15/20 (75%) preferred the icon of a patient with an adjacent intravenous pole. Among these 20 participants, 15/20 (75%) spontaneously chose the term “patient” as the preferred label. “Patient” was also the only term that wasn’t noted as being disliked or upsetting by at least one respondent from the list of potential labels (kid, child, young person, patient or teenager).

**Table 1:** Demographic Information of the Participants by Phase of Development

	Low Fidelity			Design Phase				High Fidelity		
				SSPedi Report Design		Overall Design		Coding Sprints		Finalization
	Cohort 1 (*n* = 10)	Cohort 2 (*n* = 10)	Cohort 3 (*n* = 10)	Cohort 4 (*n* = 10)	Cohort 5 (*n* = 5)	Cohort 6 (*n* = 10)	Cohort 7 (*n* = 5)	Cohort 8 (*n* = 10)	Cohort 9 (*n* = 10)	Cohort 10 (*n* = 10)
Participant Age in Years										
8–10	0	4	2	3	1	3	2	2	3	2
11–14	8	3	3	6	2	5	1	6	4	5
15–18	2	3	5	1	2	2	2	2	3	3
Participant Male	5	6	6	6	4	7	3	8	5	4
Inpatient	10	9	9	5	2	6	3	6	6	5
Reason for Visit										
Chemotherapy	8	9	6	9	4	7	4	9	6	8
Other	2	1	4	1	1	3	1	1	4	2
Cancer Diagnosis										
Leukemia or lymphoma	6	9	7	7	3	8	2	4	8	1
Solid tumor	2	1	2	2	2	2	2	4	2	8
Brain tumor	1	0	1	1	0	0	1	0	0	1
Other	1	0	0	0	0	0	0	2	0	0

### Low-fidelity phase

Table 2 shows the results of low-fidelity development by cohort. Understanding (rated as mostly correct or completely correct) was high (80–100%) for all tasks, pages, icons, and reports. Minor edits to the pages such as text size, figures and icon placement were made based upon qualitative comments, resulting in a final version that was considered satisfactory by the Review Panel after inclusion of 30 participants. Among the entire 30 respondents included in low-fidelity development, 28 (93.3%) found the SPARK website easy or very easy to use, 24 (80%) found SPARK reports easy or very easy to understand and 26 (86.7%) thought SPARK would be useful or very useful for future pediatric patients receiving cancer therapies.

**Table 2:** Number Mostly or Completely Correct During Low and High-Fidelity (Finalization) Phases of SPARK Development by Task^a^

Task	Low Fidelity			High Fidelity Finalization
	Cohort 1 (*n* = 10)	Cohort 2 (*n* = 10)	Cohort 3 (*n* = 10)	Cohort 10 (*n* = 10)
Ability to Perform Specific Functions				
Completing SSPedi	NA	9 (90%)	10 (100%)	10 (100%)
Seeing current or previous SSPedi scores	NA	10 (100%)	10 (100%)	10 (100%)
Landing Page				
Overall	8 (80%)	9 (90%)	10 (100%)	10 (100%)
Child icon	10 (100%)	10 (100%)	10 (100%)	10 (100%)
Family member icon	10 (100%)	10 (100%)	10 (100%)	10 (100%)
Healthcare provider icon	10 (100%)	10 (100%)	10 (100%)	10 (100%)
What are SPARK and SSPedi	9 (90%)	9 (90%)	10 (100%)	10 (100%)
Do SSPedi Now!	10 (100%)	10 (100%)	9 (90%)	10 (100%)
How Will SSPedi Help Me?	9 (90%)	8 (80%)	8 (80%)	10 (100%)
My SSPedi Scores				
Single SSPedi administration report	10 (100%)	10 (100%)	10 (100%)	10 (100%)
Navigating to report of a specific symptom over time	10 (100%)	9 (90%)	10 (100%)	10 (100%)
Interpreting specific symptom improving over time	9 (90%)	9 (90%)	10 (100%)	10 (100%)
Interpreting specific symptom worsening over time	9 (90%)	9 (90%)	9 (90%)	10 (100%)

NA - not evaluated in Cohort 1 as need to test was first identified at the first Review Panel meeting

^a^As assessed by a two interviewers rated on a 4-point Likert scale ranging from completely incorrect to completely correct

### Design phase

There were 15 respondents included in the SSPedi report design sub-phase in which the single SSPedi administration report (with all 15 symptoms) was evaluated, and 15 respondents in the overall design sub-phase in which the five developed pages were evaluated. For the first 10 patients in the SSPedi report design sub-phase (cohort 4), 7 (70%) preferred the single SSPedi administration report to be laid out with horizontal symptom score bars such that different symptoms were presented along the Y-axis (Fig. 5). Similarly, 7 (70%) felt this orientation made the symptom report easier to understand. Consequently, this orientation was used in future testing. Next, we identified the preferred colors for the multi-colored version of the single administration SSPedi report using the 10 children in cohort 4, which resulted in the following color scheme: green (not at all botherered), yellow (a little), light orange (medium), dark orange (a lot) and red (extremely bothered). In the comparison of multi-colored versus monotone symptom bars, 9 (90%) preferred the multi-colored version and 7 (70%) said this version made the symptom report easier to understand. Consequently, the multi-colored version was taken forward for future testing. Using the 5 children in cohort 5, we found that all 5 (100%) were correctly able to understand the revised SSPedi single administration report. Among the 15 children included in the overall design sub-phase, all understood the SPARK landing page, the “Do SSPedi Now!” page, the SSPedi single administration report, and the change in a symptom-over-time report, while 14 (93.3%) understood the “How Will SSPedi Help Me?” page. Preferences for pages, icons, pictures and colors led to subsequent refinements of SPARK until this phase was considered complete by the Review Panel with the inclusion of 30 participants total.

### High-fidelity phase

During high-fidelity development, there were 20 respondents included in the coding sprints. We found that 20 (100%) understood how to access and complete SSPedi, 19 (95%) were able to retrieve SSPedi instructions, 20 (100%) understood how to cancel out of SSPedi and 20 (100%) understood how to save scores. Based upon qualitative comments, minor edits were made to the SPARK pages leading to the finalization sub-phase. Understanding (rated as mostly correct or completely correct) was perfect (100%) for all tasks, pages, icons and reports in cohort 10 (Table 2). Among these respondents, 10 (100%) found the SPARK website easy or very easy to use and 9 (90%) found the SPARK reports easy or very easy to understand. All 10 (100%) thought the website would be useful or very useful for future patients receiving cancer therapies.

## Discussion

We used an iterative and phased approach with cognitive interviewing and quantitative and qualitative feedback from children receiving cancer treatments to develop, evaluate, refine and finalize the patient-specific aspects of the SPARK website. Allowing the target population to directly influence each element of SPARK should improve the usability and usefulness of SPARK from the perspective of children with cancer and pediatric hematopoietic stem cell transplant recipients. We found this approach allowed participants to focus on individual aspects, for example navigation or design, and provided valuable feedback on distinct elements. This approach to measuring user preferences may be useful for other health app developers. A novel aspect of this study was the use of a Review Panel with patient representation and inclusion of a behavioral scientist specifically focused on choice architecture to enhance SPARK design and useability.

While we were sucessful in this initiative, this study was relatively resource intensive and required the enrollment of 90 children and two interviewers to conduct all evaluations. We found that using a second interviewer was important when performing cognitive interviews as it allowed one person to engage with the child and not be concerned with record keeping or other administrative tasks.

An important aspect of this study was the use of InVision to minimize the costs of SPARK development. InVision is a digital design platform that allowed us to create an interactive mock version of SPARK. Using InVision in low-fidelity development made it easier, faster and less expensive to edit the web pages based upon the early responses until the web pages were relatively refined. It was only these refined web pages that were then hard coded by the website developers for the coding sprint sub-phase. We believe that this approach could be used more generally to create web applications that require user feedback to improve design and function.

The strengths of our study include its step-wise and phased approach to SPARK development and evaluation. We also included direct testing of different versions of draft web pages in a side-by-side approach, which allowed children to identify a preferred version. We successfully showed how we could use pre-existing methods and using the Technology Acceptance Model, engage with a pediatric population with a serious medical condition to refine a health technology. Since SPARK is ultimately intended toward this population, it was important that understandability, usability and usefulness be evaluated from their perspective.

However, this study is limited by its conduct at a single center and only with English-speaking children. These aspects hinder the generalizability of our findings. Enrollment of participants from different centers would have improved generalizability but would have made the iterative evaluative process more logistically complex. Another limitation of this study is the time and resources required to complete each phase of testing. Also, these results are applicable to only this one product, namely SPARK. While the results are not directly transferable to other settings, understanding our evaluative approach and iterative design may be useful to other researchers wishing to create similar products for the pediatric population. Finally, further evaluation of the refined version of SPARK would be useful and we intend to include further qualitative evaluations during longitudinal evaluation to identify whether further modifications would be beneficial.

## Conclusion

In conclusion, SPARK is a web-based application that is usable and understandable from the perspective of children aged 8 to 18 years receiving cancer therapies. It is now appropriate to conduct research using SPARK, including longitudinal administration of SPARK to children or adolescents receiving cancer treatments. Future efforts should focus on clinical implementation of SPARK.

## Availability and requirements

**Project Name:** SPARK (Supportive care Prioritization, Assessment, and Recommendations for Kids).

**Project home page:** https://www.sungresearch.com/spark/

**Operating system:** Platform independent.

**Other requirements:** NA.

**Programming language:** Java.

**License:** NA.

**Any restrictions to use by non-academics:** Currently only available for use in research through The Hospital for Sick Children.

## Abbreviations

SPARK: Supportive care Prioritization, Assessment and Recommendations for Kids
SSPedi: Symptom Screening in Pediatrics Tool
